# Biostable ssDNA Aptamers Specific for Hodgkin Lymphoma

**DOI:** 10.3390/s131114543

**Published:** 2013-10-25

**Authors:** Parag Parekh, Sanchit Kamble, Nianxi Zhao, Zihua Zeng, Jianguo Wen, Bin Yuan, Youli Zu

**Affiliations:** Department of Pathology and Genomic Medicine, Houston Methodist Hospital, and Cancer Pathology Research Laboratory, Houston Methodist Research Institute, Houston, TX 77030, USA; E-Mails: pparekh@houstonmethodist.org (P.P.); skamble@houstonmethodist.org (S.K.); nzhao@houstonmethodist.org (N.Z.); zzeng@houstonmethodist.org (Z.Z.); jwen@houstonmethodist.org (J.W.); byuan@houstonmethodist.org (B.Y.)

**Keywords:** aptamer, biostability, Hodgkin lymphoma, single-stranded DNA (ssDNA), tumor cell detection

## Abstract

As a “chemical antibody”, oligonucleotide aptamers can specifically bind to their target molecules. However, clinical potential of aptamers in disease diagnosis is not yet fully explored. Using a tumor cell-based selection protocol, we developed single-stranded DNA aptamers for Hodgkin lymphoma (HL) tumor cells. The aptamers specifically bound to HL cells with a high affinity, reaching maximal cell binding at 10 nM final concentration. Importantly, the aptamers were able to selectively detect HL cells and did not react to other tumor or blood cells in mixed samples, indicating that the aptamers can be used as a specific probe for *in vitro* analysis of HL cells. Moreover, due to the inherent properties of DNA, the aptamers were stable in human serum, suggesting potential for *in vivo* detection of HL tumor cells.

## 1. Introduction

Clinical management of lymphoma is highly dependent on classification and grouping of lymphoma based on the morphology and immunophenotyping [1,2].The next-generation sequencing technologies and gene expression profiles have not yet made a significant contribution towards the determination of new molecular subtypes or categories of lymphoma [3–6]. The latest classification of lymphoid neoplasms was updated by the World Health Organization in 2008 [7]. Lymphoid neoplasms recognized by the International Lymphoma Study Group are classified in three major groups, namely, B-cell neoplasms, T-cell neoplasms, and Hodgkin lymphoma [8,9]. These groups, in turn, consist of multiple distinct clinicopathological entities, such as follicular lymphoma, Burkitt lymphoma, anaplastic large cell lymphoma (ALCL), *etc.*, based on their immunophenotype, and molecular and clinical features [10]. This current classification also allows for gray-zone or borderline lymphomas with features of both Hodgkin and non-Hodgkin lymphoma (NHL) [11–14]. These borderline cases provide an example of overlapping biology between HL and NHL [15,16]. Immunophenotyping lymphomas for appropriate treatment regimens and prognosis are usually done by immunohistochemistry (IHC) or flow cytometry (FCM) [17–19]. Selection of the right antibody panels is of utmost importance when phenotyping cells. Aptamers provide a new toolbox for phenotyping lymphomas with many advantages over antibodies.

Aptamers have high specificity and affinity towards their target and have been generated for any given molecular target ranging from small molecules, such as ions, drugs, and toxins, to large molecules, such as peptides, proteins, and even whole cells, bacteria, and viruses [20–26]. Aptamers are composed of DNA or RNA oligonucleotides and are considered “chemical antibodies” meaning, in addition to the certain advantages, such as high affinity and specificity, chemical synthesis, long-term storage, and ease of modification, they also provide an economic and a superior alternative to antibodies. In addition, aptamers show minimal or no immune response *in vivo* [27,28].

Polymerase chain reaction was developed in 1983 and the first aptamers were reported in 1990 [29]. SELEX is an acronym for Systematic Evolution of Ligands via Exponential Enrichment, the process used for the *in vitro* procedure to select aptamers for a particular target [30,31]. It is an iterative process consisting of panning out binders and non-binders from a library consisting of approximately 10^14^–10^15^ unique random sequences. This separation is achieved by different methods, such as the use of membranes, columns, magnetic beads, and capillary electrophoresis [23,32,33]. Advances in SELEX methodology include the modifications, such as the inclusion of counter selection, toggle selection, and photo-SELEX to maximize affinity and specificity or impart additional unique properties to the selected aptamer [34].

Aptamers have also been used for phenotyping cells. We have demonstrated in our previous studies that CD4 and CD30 aptamers can be used for immunophenotyping cells [35,36]. In the current study, we used SELEX to generate aptamers that specifically detect HL cells and, therefore, distinguish them from other lymphoma cells, allowing for the aptamers to be used in cell phenotyping. The ssDNA-based aptamer has higher biostability in comparision with similar RNA-based aptamers due to the inherent stability of DNA bases over RNA and, hence, can be advanced for *in vivo* therapeutic applications. Since the aptamer recognizes molecular level differences on the live cell surface, this HL-targeting aptamer can not only be used for phenotyping, but also for targeted drug delivery to HL cells. The aptamer was highly sensitive and shown to detect HL cells in a mixture of cells and even complex biological medium, such as whole blood.

## 2. Experimental Section

### 2.1. Cell Lines and Reagents

HDLM2 and KM-H2 (Hodgkin lymphoma), Karpas-299 (K299) and SU-DHL-1 (anaplastic large cell lymphoma), U937 (histiocytic lymphoma), Jeko-1 (B-cell lymphoma), Maver-1 (mantle cell lymphoma), CA46 (Burkitt lymphoma), K562 (chronic myeloid leukemia), HL60 (acute promyelocytic leukemia), Jurkat and H9 (T-cell lymphoma), MM1S (multiple myeloma), and LNCAP (prostate adenocarcinoma) cell-lines were cultured in Roswell Park Memorial Institute (RPMI)-1640 medium; a SKBR3 (breast adenocarcinoma) cell line was cultured in McCoy's 5a modified medium; and a HEK293 (human embryonic kidney) cell-line was cultured in Eagle's Minimum Essential Medium (EMEM) medium. The media were supplemented with heat-inactivated Fetal Bovine Serum (FBS) (GIBCO, Grand Island, NY, USA) and 100 IU/mL penicillin-streptomycin, and were incubated at 37 °C in a 5% CO_2_ atmosphere. All of the cell lines were obtained from American Type Culture Collection (ATCC, Manassas, VA, USA).

Dulbecco's PBS (Sigma, St. Louis, MO, USA) enriched with 4.5 g/L glucose and 5 mM MgCl_2_ was used as washing buffer during selection and flow cytometry assays. Binding buffer comprised of washing buffer containing 1 mg/mL BSA (Fisher, Waltham, MA, USA) and 0.1 mg/mL t-RNA was used to reduce nonspecific background binding.

### 2.2. SELEX Primers and DNA Library

The ssDNA library for SELEX contained a random core of 30 mer flanked with an 18 mer primer binding region on both sides. A biotinylated reverse primer 5′- GTC AAC CGA ATG CGT CAG -3′ was used to generate single-stranded DNA, and a fluorophore (either FITC or Cy5)-labeled forward primer 5′- TAC CAG TGC GAT GCT CAG -3′ was used to monitor the progress of aptamer selection. The primers were designed with OligAnalyzer® software from IDT Technologies. The aptamer pools were PCR amplified with Taq polymerase and PCR reagents from Takara Bio (Mountain View, CA, USA).

### 2.3. Cell-Based SELEX

CD30-positive HDLM2 cells were counted with a hemacytometer and two million cells were washed with PBS, centrifuged at 270 g, and used for SELEX. The cells were incubated with a DNA library, which was rapidly cooled on ice after heating at 95 °C for 5 min. Selection was initiated with a 250 nM ssDNA library that showed a similar preference for CD30-positive cells, including HL cell line HDLM2 and ALCL cells (K299) from a previous selection [37]. The HDLM2 cells were used for positive selection with increasing stringency by reducing the incubation time from 60 min in the first round to 30 min at the end of selection (16 rounds of selection). Unbound DNA was removed by centrifugation, and the target-bound DNA eluted by heating the cells at 95 °C for 5 min. Counter selection with CD30-positive ALCL K299 cells, performed after every four rounds of positive SELEX, was introduced to reduce the number of aptamers that bound to the commonly expressed proteins and other molecular markers on the surface of other related lymphoma cells that were CD30 positive. The eluted DNA was amplified by PCR, and PCR conditions were optimized with *Taq* polymerase to yield a clear, single band after each round of SELEX. High-affinity streptavidin-sepharose beads were used to capture the biotinylated anti-sense strand, and the sense strand with the flurophore was eluted using 200 mM NaOH. The resulting ssDNA was used for the next round and the process was repeated iteratively until significant affinity towards target HDLM2 cells was observed using flow cytometry. The stringency of selection was increased by reducing the concentration of the aptamer pool used for selection and time of incubation, and increasing the washing time and volume. The resulting family of aptamers was specific to the target cells, with only a few minimal off-target aptamers. We tested the selection progress with flow cytometry using Cy5-labeled primers to generate a fluorescently-labeled pool with CD30-positive and -negative cells. The selection was stopped when no further progress was observed for 3 rounds of selection.

### 2.4. Sequencing of DNA Aptamers

Pools 13 and 16 were tagged with the multiplex identifier primers for next-generation sequencing. Fusion PCR was performed with Ion Torrent Primers; primer A was fused with the SELEX forward primer and the Ion torrent primer trP1 was fused to the SELEX reverse primer. After optimization of fusion PCR, the products were purified by size separation using TBE-urea gels, recovered using Gen-Elute PCR purification kits from Sigma, and sequenced using the Ion-Torrent sequencing platform. Analysis of NGS data was performed using MAFFT alignment software [38,39].

### 2.5. Flow Cytometric Analysis

The aptamer pools were tested using 200 nM of the enriched pool to monitor SELEX. One-half million HL HDLM2 cells were washed with PBS, incubated in a 100 μL binding buffer for 1 h, washed with 5× volume of washing buffer, and then re-suspended in 250 μL binding buffer. Ten thousand cells were counted on a BD Fortessa flow cytometer (Becton Dickinson Immunocytometry Systems, Franklin Lakes, NJ, USA). The fluorescence readouts represented the affinity of the fluorophore-labeled pools towards the HHLM2 cells. Similarly, different lymphoma cells were used for flow binding assay. After the generation of aptamers, FITC-labeled aptamers were used for characterization using different types of lymphoma and non-lymphoma cancer cell lines. The apparent dissociation constants (app Kds) were measured by flow cytometry using a series dilution of the aptamers that was incubated and analyzed on the flow cytometer.

### 2.6. Competition Experiments

Competition experiments were performed by using one unlabeled aptamer, PS1 or the truncated aptamer PS1NP shortened by the removal of primer sites, in excess (10×). Competition of PS1 and PS1NP aptamers with CD30 aptamer C2NP and CD30 antibody was done in presence of excess unlabeled aptamers. The experiment was performed by incubating unlabeled C2NP in 10× excess with Cy3-labeled PS1 or PS1NP for 1 h. PE-labeled CD30 antibody was used to compete with unlabeled PS1 and PS1NP.

### 2.7. Biostability Assays

One microgram of DNA aptamers was incubated with human serum for 0, 2, 8, and 24 h. These samples were subjected to the standard phenol-chloroform DNA extraction protocol and the recovered and digested aptamers were visualized on a 5% agarose (TBE) gel. Fluorescence was quantified using the densitometry tool in the Bio-Rad Image Lab software.

### 2.8. Aptamers for Cell Phenotyping and Detection in Complex Media

Target HDLM2 cells were mixed with non-target U937 cells and incubated with 5 nM of Cy3-labeled aptamers for 1 h, washed with 5× washing buffer, and tested on a flow cytometer. Specific binding of 5 nM of aptamers was also tested by addition of HDLM2 cells with CD30-positive K562 cells. Similarly, HDLM2 cells were mixed with whole blood and tested with 5 nM of aptamers PS1 and PS1NP with flow binding assays for specific detection of HL cells in whole blood.

### 2.9. Fluorescence Microscopy

Imaging was performed using an Olympus epifluorescence microscope (Olympus America, Center Valley, PA, USA). HDLM2 cells, U937 cells, and a mixture of HDLM2 cells and U937 control cells were incubated with 100 nM of Cy3-labelled aptamer PS1 for 1 h, and then washed with 5× washing buffer and resuspended in 100 μL binding buffer for imaging.

### 2.10. Statistics

Standard deviation is represented by error bars in the graphs showing the binding affinity experiments which used the average of the readings from two different experiments in duplicate. The graphs were plotted using MS-Excel 2010, and the integral Solver toolpack was used for data analysis.

## 3. Results and Discussion

### 3.1. Development of Single-Stranded DNA Aptamers with High Affinity to Hodgkin Lymphoma Tumor Cells

**Figure 1.**
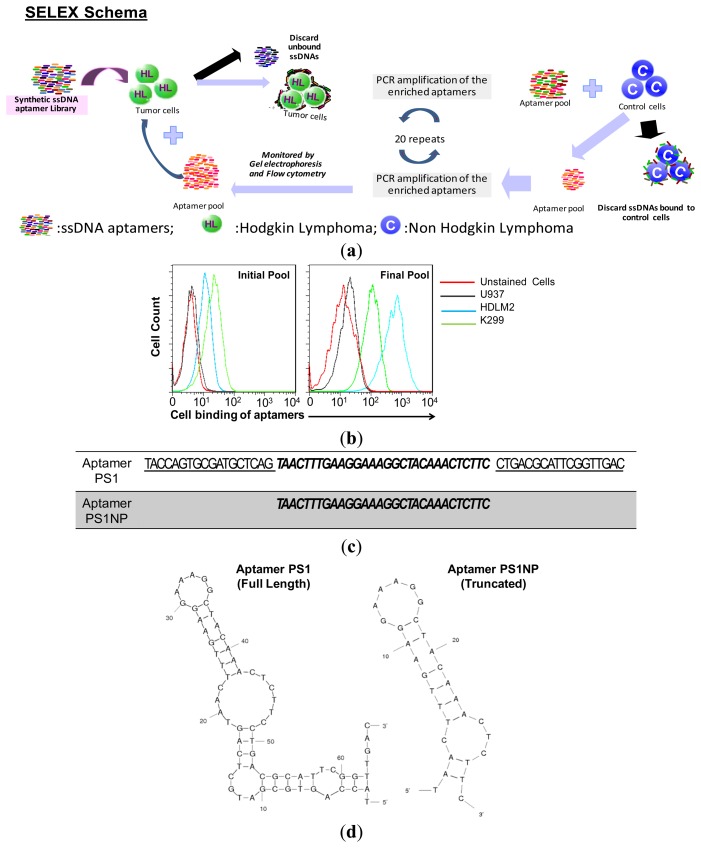
Selection, identification, and optimization of aptamers specific for HL tumor cells. (**a**) SELEX scheme for selection of aptamers; (**b**) a pool enriched for CD30-positive cells was used to select an aptamer that will bind Hodgkin lymphoma cells with high affinity. The left panel shows the affinity of the initial pool towards HL cells (HDLM2) and ALCL cells (K299). The final pool shows the enrichment of aptamers that recognize HL cells with higher affinity, compared to other lymphoma cells; (**c**) individual aptamer sequence PS1 and its truncated form, PS1NPD; (**d**) secondary structure prediction as revealed by mFold software depicting the stem-loop structure that is maintained even after the removal of primers as the probable minimal binding region of aptamer PS1.

Lymphomas are classified as Hodgkin (HL) and non-Hodgkin lymphoma. Most HL originate from germinal center B-cells and a rarely from T-cells [40,41]. Cell-surface expression of CD30 is a marker for Hodgkin disease [42–44]. However, some T-cell lymphomas, especially ALCL, mimic HL by cell surface expression of CD30 [45,46]. With this insight, we aimed to isolate CD30-independent aptamers for HL cells. A pool, that included ALCL cells with a similar affinity for CD30-positive HL, was used to select aptamers specific only for HL cells. The intention was to identify aptamers that bind HL with high affinity, but do not bind CD30 and other lymphoma cells. This initial pool had a slightly higher affinity for CD30-expressing ALCL cells when compared with CD30-expressing HL cells. This pool contained a 30 mer random region flanked by primer sites on both ends. The progress of SELEX was monitored by flow cytometry to test the binding of the enriched pool towards the HL and NHL cells. Figure 1a depicts the SELEX scheme used for the selection of aptamers. In Figure 1b, the left panel shows the affinities of the pool at the start of selection and the right panel shows the affinity of the pool at the end of selection. The results show that the aptamer pool is highly enriched for HL cells, with moderate affinity for ALCL cells, but no affinity for other lymphoma cells. The ssDNA pool was sequenced after 13 and 16 rounds of enrichment and selection using the cell-based SELEX method.

Next-generation sequencing was used to sequence different pools tagged with an identifier sequence. Among the aptamer sequences, sequence PS1 (Figure 1c) showed the highest frequency, represented 14.2% of all sequences in pool 13, and increased its contribution to 18.1% in the final pool 16 as SELEX progressed and, hence, was selected for further analysis. To optimize aptamer PS1, a series of truncated sequences were analyzed in an attempt to achieve a minimum binding region that improved or maintained the binding affinity and specificity. The secondary structure of original and sequence-trimmed aptamers was visualized using mfold, and is shown in Figure 1d [47,48]. Secondary structures, as revealed by the software, demonstrated that the trimming of either primer on each site altered the secondary structure, yet both aptamers retained a part of the parent stem-loop structure. The core sequence of the PS1 aptamer without primer sequences on both sides also contained a stem-loop structure similar to the parent sequence, which was then synthesized, and the resulting truncated aptamer was designated PS1NP (Figure 1d, right).

### 3.2. ssDNA Aptamers Specific for HL

**Figure 2.**
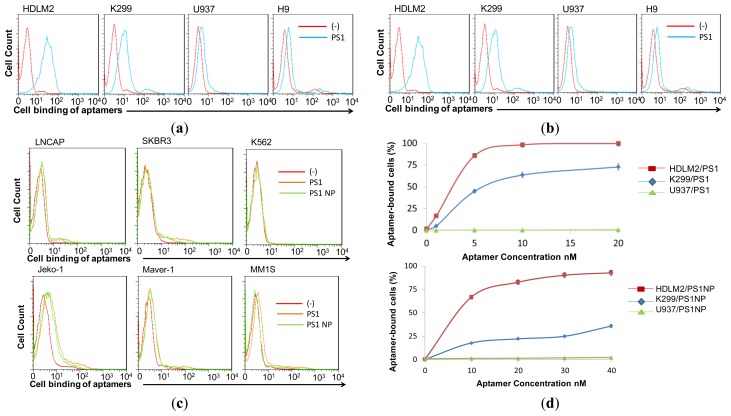
Specific binding of aptamers to HL tumor cells. (**a**) Aptamer PS1 and (**b**) PS1NP bind HL cells (HDLM2), ALCL cells (K299), and other lymphoma cells (U937 histiocytic lymphoma and H9 T-cell lymphoma cells); (**c**) Aptamers PS1 and PS1NP do not bind different control tumor cell lines: Jeko-1 and Maver-1 B-cell lymphoma, MM1S multiple myeloma, SKBR3 breast adenocarcinoma, LNCAP prostate adenocarcinoma, and K562 chronic myeloid leukemia cells; (**d**) Apparent dissociation constants (app. Kds) of aptamers PS1 and PS1NP with HDLM2, K299, and non-target U937 cell-lines.

To rule out potential adverse effects of sequence shortening, specificity of aptamer PS1NP was validated by cell-binding analysis along with the ssDNA aptamer PS1. Both the full length and the trimmed sequence were synthesized with their 5′ end conjugated with a Cy3 fluorescent reporter. The aptamer probes were incubated with cultured HL tumor cells (HDLM2 and KMH2), ALCL tumor cells (K299 and SUDHL1), other lymphoma cells (U937, H9, Jurkat, CA-46, Maver-1, and Jeko-1), and other tumor cell lines (K562, MM1S, HL-60, SKBR3, LNCAP, and HEK293). After treatment for 1 h at room temperature, resultant cell binding of aptamers was quantified by flow cytometry analysis. As shown in Figure 2a,b, both aptamer sequences specifically bound to HL cells, but did not react to other tumor cell lines (Figure 2c). The aptamers also showed minimal binding towards ALCL cells expressing CD30, but did not bind other cell lines expressing CD30, implying a CD30-independent binding to the cells. Since PS1 and PS1NP showed similar binding patterns, this confirmed that PS1NP did not lose its specificity during truncation. Table 1 shows the binding affinity of the aptamers towards the different tumor cell lines grouped as non-binding, weak and strong binders.

In addition, cell-binding affinity of aptamers was analyzed using a series dilution. Flow cytometry dose course assays revealed that aptamer PS1 had a high binding affinity and an apparent Kd of 5 nM ± 0.5 nM. The short aptamer PS1NP reached its maximal binding affinity at 10 nM ± 0.6 nM, indicating a slight loss of affinity due to the truncation when compared with the full-length aptamer (Figure 2d).

### 3.3. HL Cell Binding of Aptamers was CD30-Independent

**Figure 3.**
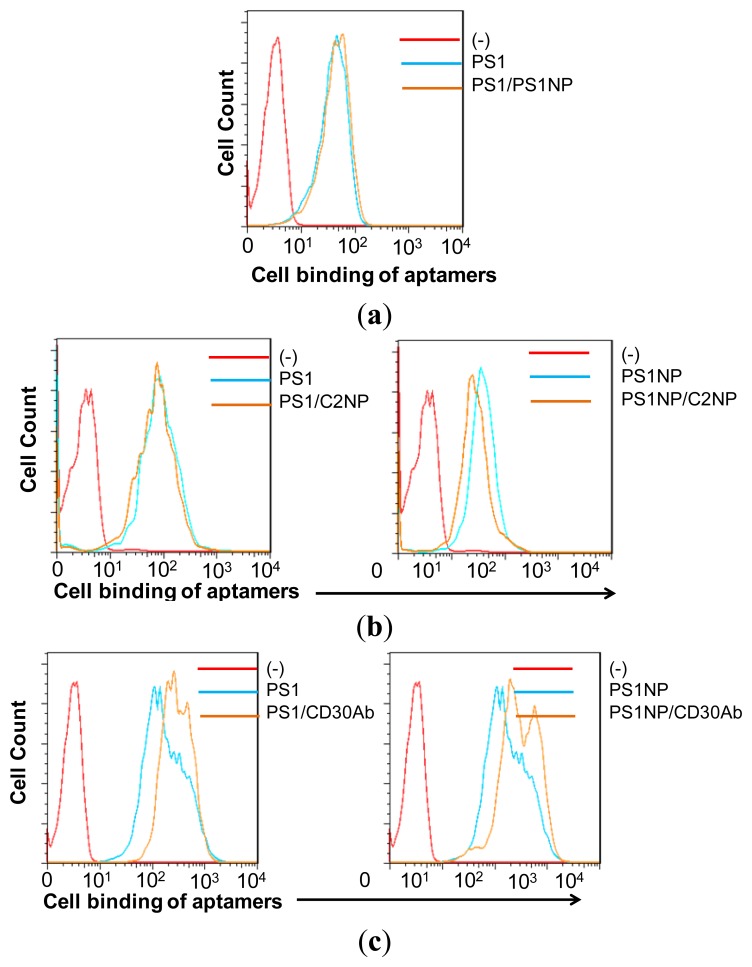
CD30-independent binding of aptamers to HL tumor cells. (**a**) The full-length aptamer PS1 and shorter, unlabeled aptamer PS1NP were incubated with HDLM2 cells and tested with flow cytometry. The presence of 10× unlabeled aptamer PS1NP did not affect the binding of PS1 and vice versa, 10× unlabeled PS1 did not impact the binding of PS1NP indicating that the short and full-length aptamer bind different epitopes of their target expressed on the cell surface; (**b**) Competition with 10× unlabeled CD30 DNA aptamer C2NP revealed that aptamers PS1 and PS1NP do not compete with the CD30 aptamer for binding their target cells, implying that the aptamers do not bind CD30; (**c**) Further validation of a non-CD30 target was done by competing the PS1 and PS1NP aptamers with CD30 antibody, which showed that they do not compete for binding.

Although aptamer PS1NP is a derivative sequence of PS1 and has a similar secondary structure, there is a possibility that they may compete to bind the same epitope of the target protein. A competition experiment between PS1 and PS1NP was performed which revealed that the PS1 and PS1NP did not compete with each other, implying that they may bind to different epitopes of their target protein (Figure 3a).

Flow cytometry analysis revealed that both PS1 and PS1NP specifically bound to CD30-expressing cells HDLM2 and K299, but not to other CD30-expressing K562 and H9 cell lines, indicating a CD30-independent target protein expressed on the cell surface. Multiple competition assays with CD30 aptamer and antibody were performed to rule out the possibility of CD30 as the target of aptamer PS1 and PS1NP. Flow cytometry analysis of the competition experiment, with simultaneous incubation of HDLM2 cells with 10-fold excess unlabeled CD30 aptamer C2NP and Cy3-labeled PS1 or PS1NP, revealed that the presence of unlabeled aptamer C2NP did not have any effect on the binding of either PS1 or PS1NP aptamer (Figure 3b), indicating that neither aptamer targeted the CD30 receptors on these cells. This was also confirmed by a competition experiment with the CD30 antibody, which also showed there is no competition between PS1 or PS1NP and the CD30 antibody (Figure 3c). These results indicate that the isolated aptamers PS1 and PS1NP bind a highly specific marker expressed on HL and ALCL cells, which is not CD30. It is probable that the target of these aptamers may be a non-CD30 biomarker for HL and ALCL, and this target may therefore be used to phenotype these cells. This target may also be used as therapeutic target for *in vivo* targeted drug delivery, specifically for HL and ALCL tumors not responsive to anti-CD30 treatment regimens [49].

### 3.4. Biostability of ssDNA Aptamer

**Figure 4.**
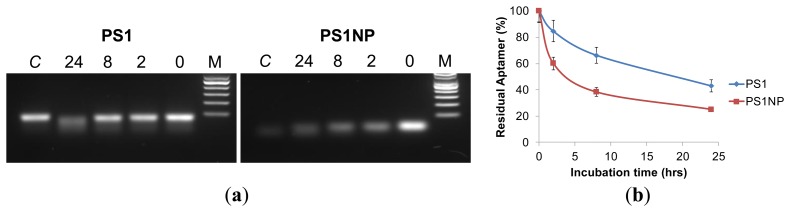
Biostability of aptamers. (**a**) Aptamers PS1 and PS1NP recovered with phenol-chloroform extraction visualized on 3% agarose gel showed that the full-length aptamer is more stable to serum nucleases when compared to the shorter aptamer; (**b**) Aptamers were separated using agarose gel electrophoresis and normalized fluorescence intensity of full-length DNA/area (mm^2^) was plotted as a function of time (hours). We observed that, even after 24 h of serum incubation, approximately 50% of PS1 and 40% of PS1NP were intact.

*In vivo* applications of aptamers require that they be stable in physiologic conditions for a substantial amount of time. Nuclease susceptibility of aptamers is one of the major hindrances in the utilization of aptamers for *in vivo* applications. The biostability of the aptamers PS1 and PS1NP was tested by the addition of the aptamers into human serum to mimic an *in vivo* physiologic condition. After incubation for 0, 2, 8, and 24 h at 37 °C, gel electrophoresis was performed with PS1 and PS1NP aptamers incubated in serum as described above. The aptamers were subsequently recovered by phenol-chloroform extraction from the serum and examined on 3% agarose gel. As shown in Figure 4a, the aptamer PS1 (left) and PS1NP (right) had minimal change from 0 to 24 h incubation in serum. Quantification of recovered DNA is depicted in Figure 4b, which also revealed that aptamer PS1 is slightly more stable than aptamer PS1NP. These findings indicate that the developed ssDNA aptamers are stable in human serum at 37 °C, a biological and physiological condition, and therefore make them suitable for *in vivo* use.

### 3.5. Aptamers for Cell Phenotyping and Detection of HL in Complex Media

**Figure 5.**
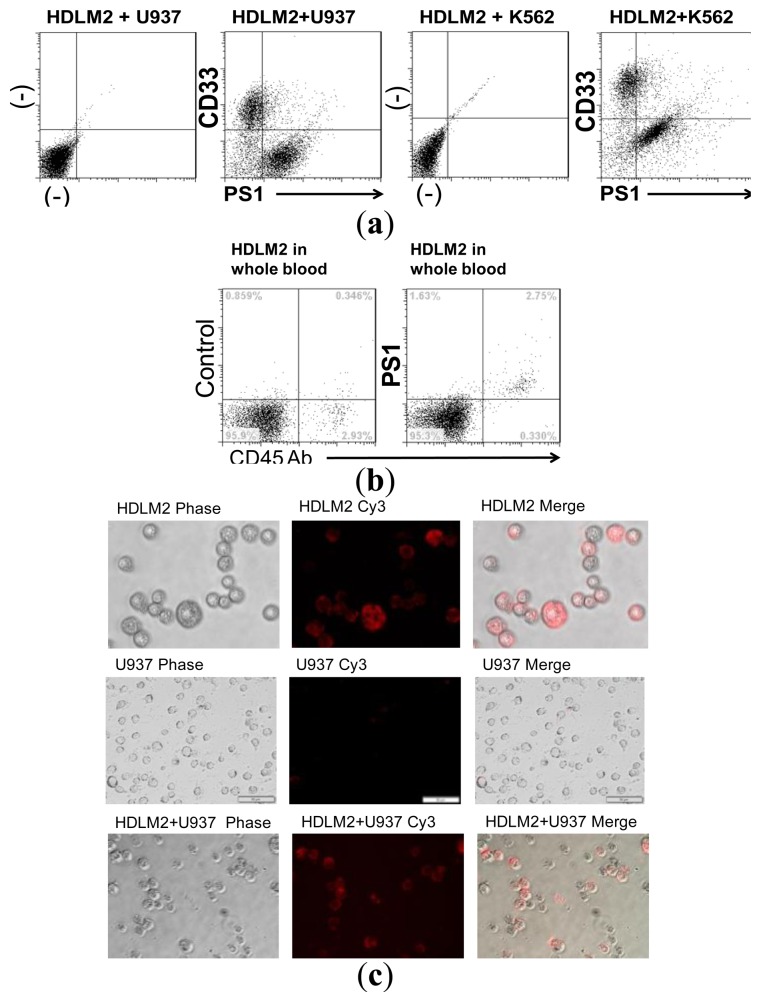
Selective detection of tumor cells by aptamers. Aptamer PS1 recognizes HL cells in mixture containing control U937 cells without non-specific binding. The aptamer also recognizes HL cells specifically in whole blood with added tumor cells. (**a**) Multicolor flow cytometry with a mixture of HDLM2 and U937 or K562 cells with aptamer PS1 at 5 nM concentration. The left panel shows unstained HDLM2 + U937 cells; the next panel shows that PS1 binds only HDLM2 cells and CD33 was used as a marker to identify control cells. Similarly, the third panel depicts unstained HDLM2 + K562 cells and the right panel with PS1 binding only HDLM2 cells; (**b**) Similarly, experiments with target tumor HDLM2 cells mixed with whole blood demonstrated that the cells were recognized by aptamer PS1; (**c**) Aptamer PS1 (100 nM) stained HDLM2 and U937 cells in a 1:1 cell mixture of HDLM2:U937 cells. The images were then captured with a fluorescence microscope. Aptamer PS1 specifically binds only the Hodgkin lymphoma cells (HDLM2).

Multiparametric flow cytometry is routinely used to immunophenotype various hematological malignancies, including HL [50–52]. Experiments were performed to demonstrate the potential of the aptamer in detection and phenotyping of HL cells. A cell mixture was made by diluting HDLM2 cells into U937 cells or K562 cells. The cell mixture was then treated with aptamer PS1and CD33 antibody that binds U937 or K562 cells. Flow cytometry analysis showed that PS1 detected only the target HDLM2 cells in the cell mixture (Figure 5a).

Furthermore, to test potential clinical use, cultured HDLM2 cells were diluted into fresh human whole blood isolated from normal donors. The tumor cell/whole blood mixture was double-stained with anti-CD45 antibody and 5 nM aptamer PS1. As shown in Figure 5b, the diluted tumor cells were highlighted by aptamer PS1. Similarly, aptamer PS1 also identified KMH2 cells in whole blood. In addition, aptamer PS1NP successfully recognized target cells only in whole blood. A cell mixture of HDLM2 cells and U937 cells in whole blood was also used to test the specificity of aptamer in complex biological fluids. Both aptamers identified only HLDM2 cells and did not bind U937 cells, even in whole blood, indicating that the aptamers can be used to phenotype target cells in whole blood specimens. Current antibody-based methods for phenotyping are not reliable in whole blood, thus aptamers provide a key advantage over antibodies in that they can be used directly with whole blood, without any processing. Selective cell staining with aptamer PS1 of HDLM2 cells, in a mixture of HDLM2 and U937 or K562 cells, was also observed by fluorescent microscope (Figure 5c), demonstrating a method for direct detection of HL or ALCL cells.

## 4. Conclusions

Aptamers have been used for various applications, such as a molecular recognition element for different analytes, as diagnostics and therapeutics, for biomarker detection and validation, *etc.* However, aptamers have not yet been widely used to phenotype cells. We describe the generation of an aptamer using cell-based SELEX that binds HL cells with high affinity with an app. Kd of 5 ± 0.5 nM. We then used the aptamer for specific cell detection in a mixture of cells in PBS and in complex biological medium, such as whole blood, thereby demonstrating the use of aptamers in cell phenotyping. The aptamer is also biostable in serum for 24 h and, hence, can be used as vehicle for *in vivo* targeted drug delivery. In conclusion, this work demonstrates the use of aptamers to detect and distinguish HL cells from other lymphoma and cancer cells using flow cytometry and fluorescence microscopy.

## Figures and Tables

**Table 1.** Summary of tumor HL cell binding of aptamers PS1 and PS1NP. The aptamers show slight binding towards ALCL cells, but not through the commonly expressed antigen CD30 on the surface of these lymphoma cells.

**Cell Line**	**Disease**	**CD30**	**PS1**	**PS1NP**
HDLM2	Hodgkin Lymphoma	+	+++	+++
KMH2	Hodgkin Lymphoma	+	+++	+++
K299	Anaplastic Large Cell Lymphoma	+	++	++
SUDHL1	Anaplastic Large Cell Lymphoma	+	+	+
K562	Chronic Myeloid Leukemia	+	-	-
H9	T-cell Lymphoma	+	-	-
Jurkat	T-cell Lymphoma	-	-	-
U937	Histiocytic Lymphoma	-	-	-
Jeko-1	B-cell Lymphoma	-	-	-
Maver-1	Mantle cell Lymphoma	-	-	-
CA-46	Burkitt Lymphoma	-	-	-
HL60	Acute Promyelocytic Leukemia	-	-	-
MM1S	Multiple Myeloma	-	-	-
SKBR3	Breast Adenocarcinoma	-	-	-
LNCAP	Prostate Carcinoma	-	-	-
HEK293	Human Embryonic Kidney	-	-	-

- no binding;

+ weak binding;

++ moderate binding and

+++ strong binding.
